# Supplementary material for: Systems Biology Approach Identifies Prognostic Signatures of Poor Overall Survival and Guides the Prioritization of Novel BET-CHK1 Combination Therapy for Osteosarcoma
Source: Cancers (Basel). 2020 Aug 26;12(9):2426. doi: 10.3390/cancers12092426 (PMC7564419; doi:10.3390/cancers12092426)
Supplement: Supplementary file 1 [file cancers-12-02426-s001.zip › Figures S1-S10 and Table S7.pdf]

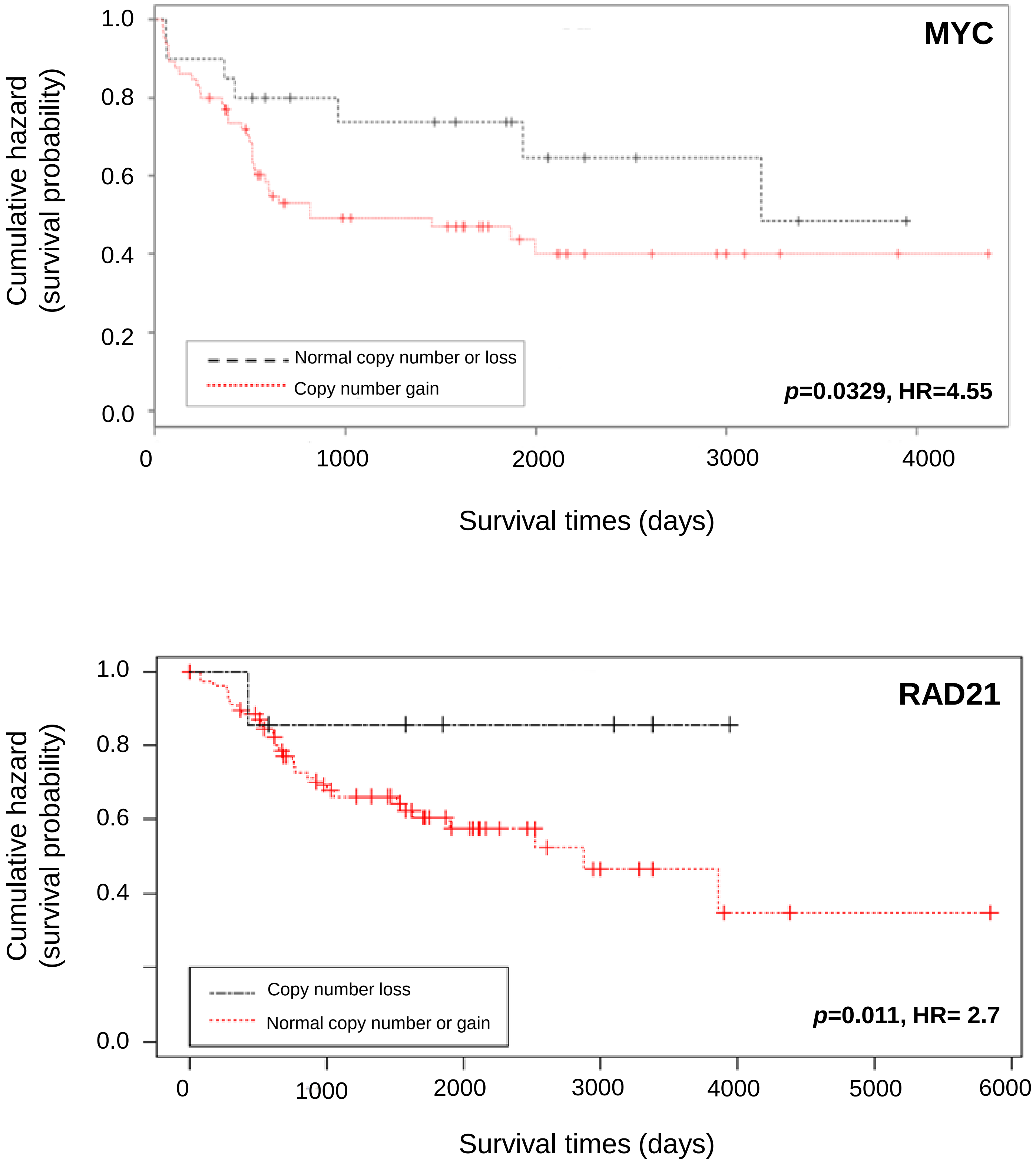

Figure S1. Alterations in MYC and RAD21 CNVs correlate with poor-survival in pediatric and AYA OS patients. (a) Kaplan Meier Survival Plot for MYC using log-rank test (p = 0.0329, HR = 4.55) from TARGET database shows that MYC copy number gain is significantly associated with increased risk for poor prognosis in OS patients. (b) Kaplan Meier Survival Plot for RAD21 using log-rank test (p = 0.011, HR = 2.70) indicates that copy number gain or normal copy number is associated with increased risk for poor prognosis in OS patients.

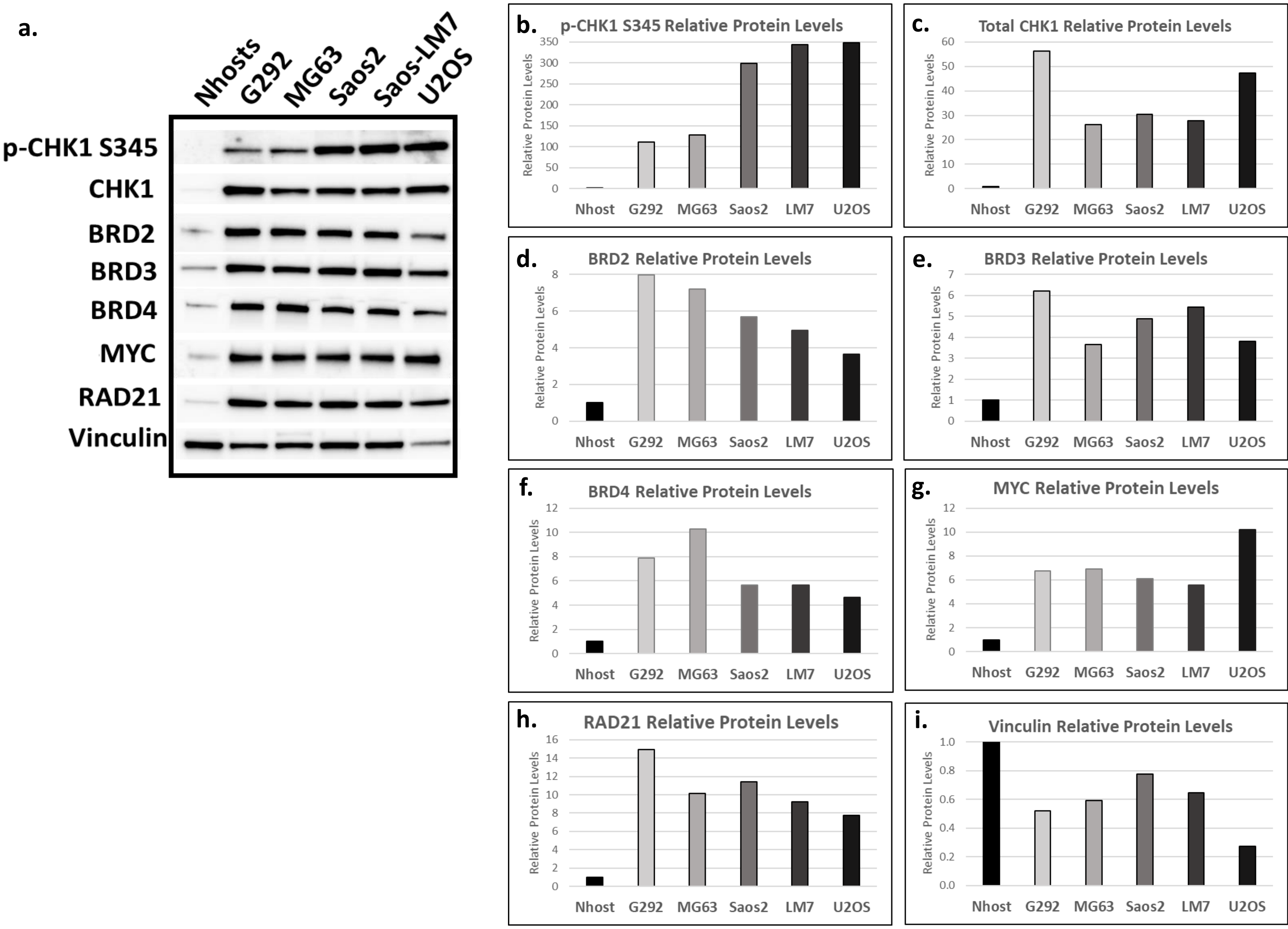

Figure S2: Validation of targets in established OS cell lines. **(a)** Western blots were conducted on whole cell lysates from five established pediatric OS cell lines to assess baseline levels of targets of interest (p-CHK1 S345, CHK1, BRD2-4, MYC, and RAD21). Vinculin served as the loading control. Normal human osteoblasts (NHOSTs) were included as the normal tissue control. **(b-i)** Image Lab software (Bio-Rad) was used for quantification of protein levels. Proteins of interest were normalized to total protein on the corresponding blot and expressed relative to control cells (NHOSTs). Compared to NHOSTs, targets of interest are expressed at higher levels in pediatric OS cell lines. This blot is representative of 2 independent experiments.

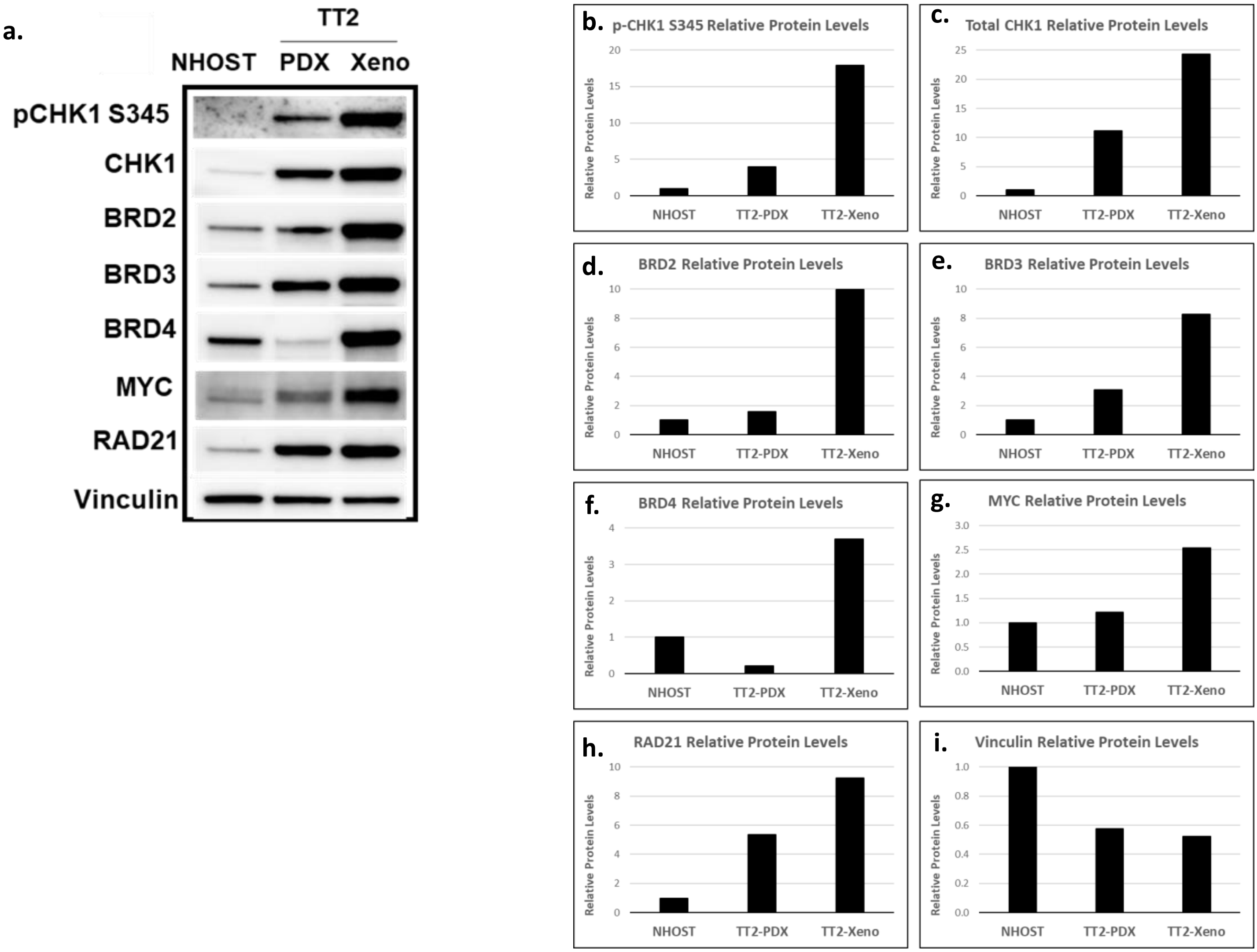

Figure S3: Validation of targets in TT2-77 PDX and PDX-derived OS Xenoline. (a) Western blot were conducted on whole cell lysates from the TT2-77 PDX as well as its xenoline to assess baseline levels of targets of interest (p-CHK1 S345, CHK1, BRD2-4, MYC, and RAD21). Vinculin served as the loading control. Normal human osteoblasts (NHOSTs) were included as the normal tissue control. (b-i) Image Lab software Bio-Rad was used for quantification of protein levels. Proteins of interest were normalized to total protein on the corresponding blot and expressed relative to control cells (NHOSTs). Compared to NHOSTs, targets of interest are expressed at higher levels in pediatric OS cell lines. This blot is from one experiment.

Table S7: MYC-RAD21 copies in established OS cell lines, TT2-77 xenoline, primary diagnostic sample, and in TT2-77 PDX samples*

Cell Lines	MYC Copies	RAD21 Copies
Saos2	5	5
Saos-LM7	3	3
MG63	12	7
G292	7	7
U2OS	4	-
TT2-77 Xenoline	3	3

TT2-77 PDX	MYC Copies	RAD21 Copies
Primary Diagnostic Block (FFPE block)	4	4
Mouse Passage 1	4	4
Mouse Passage 2	4	4
Mouse Passage 3	4	4

**Manuscript in preparation that contains whole genome sequencing data*

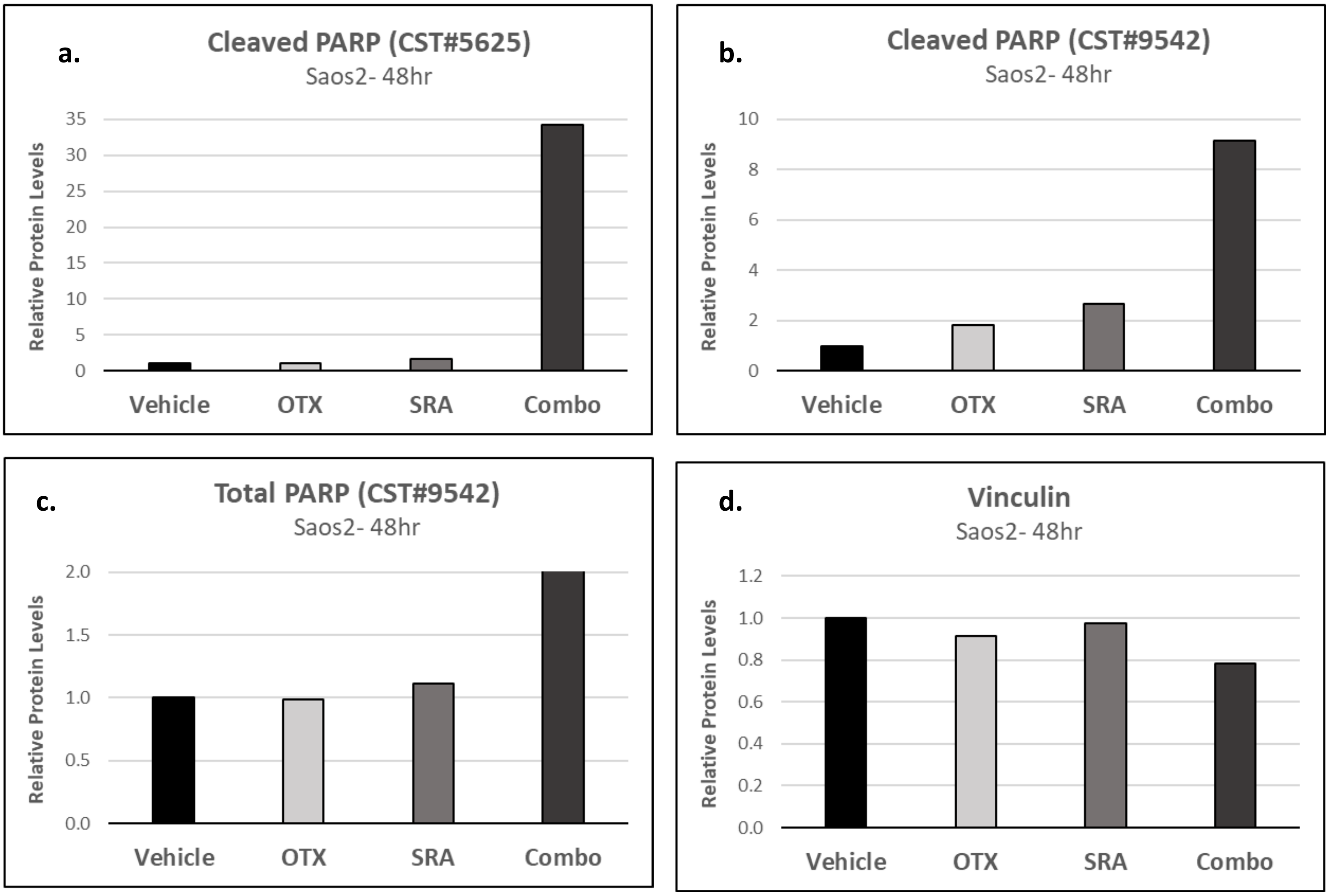

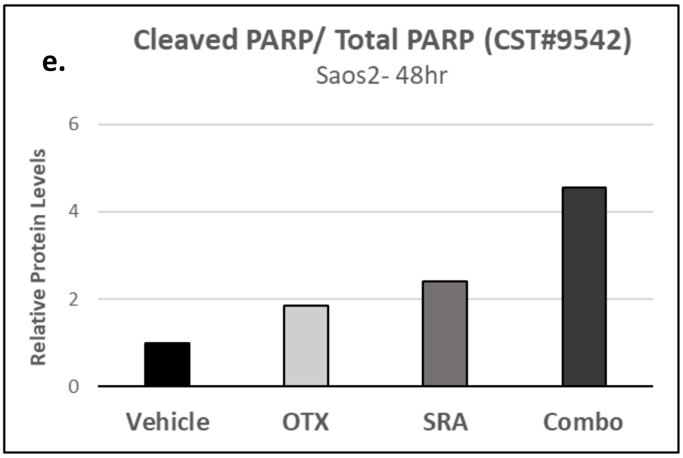

Figure S4: Quantification of cleaved –PARP protein expression in Saos2 cells treated with single agent or combination BETi + CHK1i. Proteins of interest were quantified from Figure 5b. Saos2 cells were treated with vehicle, single-agent BETi/OTX-015 (0.31µM) and/or CHK1i/SRA737 (3.75µM) for 2 days. Vinculin served as the loading control. Image Lab software (Bio-Rad) was used for protein quantification. (a-d) Proteins of interest were normalized to total protein as quantitated from the corresponding blot and expressed relative to vehicle-treated control cells. (e) Expression of cleaved PARP from Cell Signaling Technology (CST # 9542) was also normalized to total PARP protein levels. This experiment was performed one time. OTX = BETi/OTX-015, SRA = CHK1i/SRA737 , and Combo =OTX = BETi/OTX-015 + SRA = CHK1i/SRA737

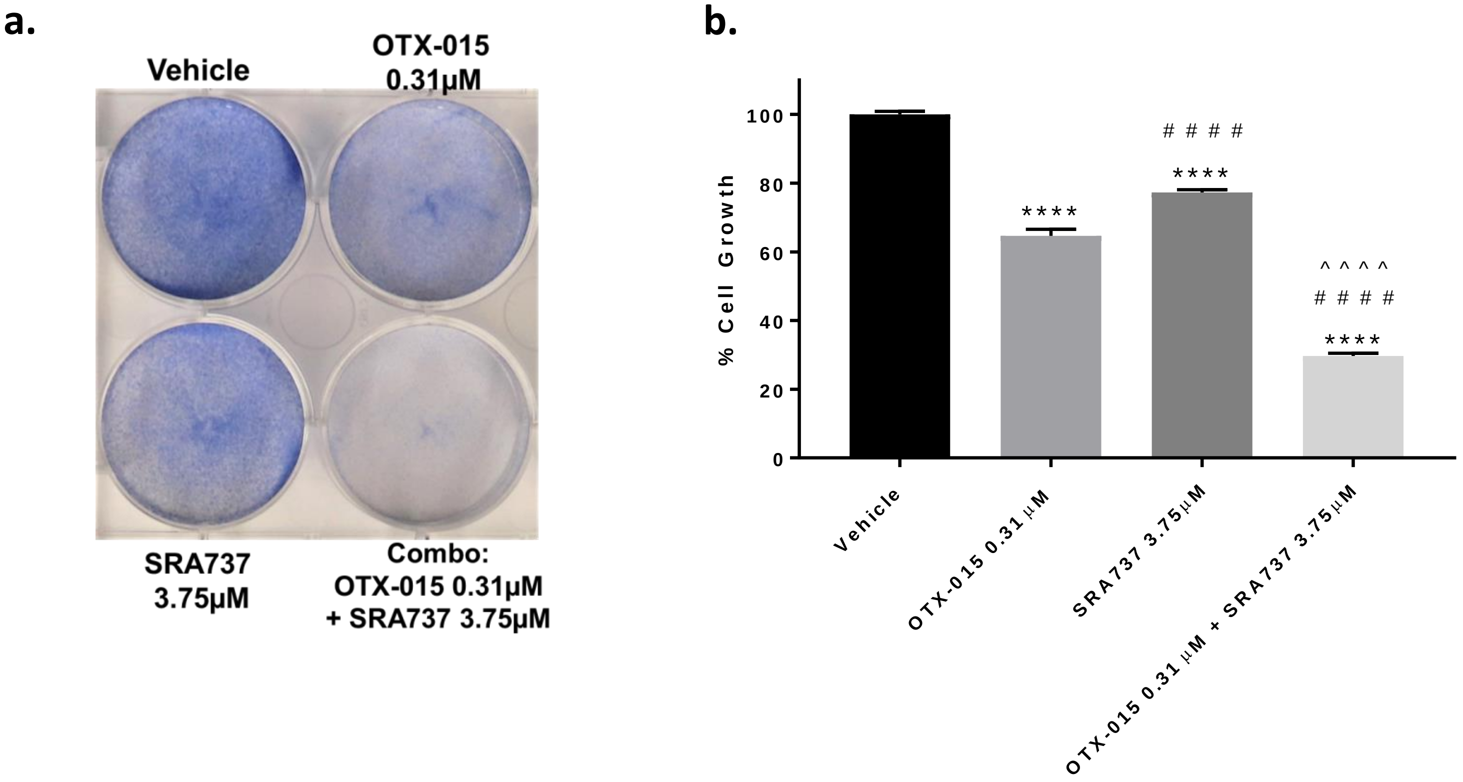

Figure S5. Combination BETi/OTX-015 + CHK1i/SRA737 inhibits cell growth in OS cells. **(a)** Saos2 cells were treated with vehicle, BETi/OTX-015 (0.31μM) and/or CHK1i/SRA737 (3.75μM) for 5 days and stained with methylene blue. **(b)** Quantification of methylene blue staining to determine % cell growth of Saos2 cells after 5 days of treatment. One-way ANOVA followed by a Holm-Sidak post-hoc pairwise multiple comparisons test, n=3 replicates per group; ****$p<0.0001$ vs vehicle; #### $p<0.0001$ vs BETi/OTX-015; ^^^^ $p<0.0001$ vs CHK1i/SRA737.

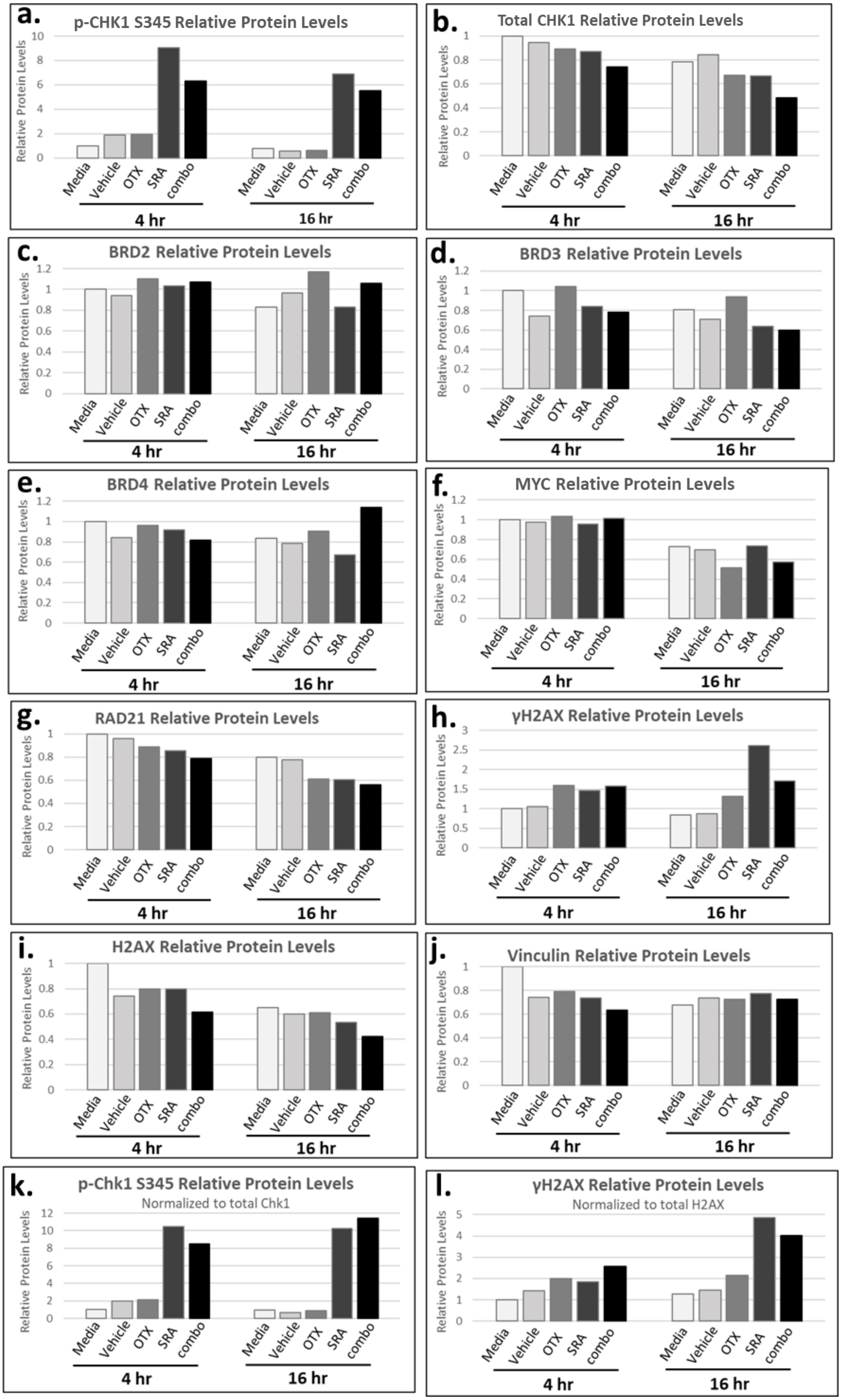

Figure S6: Quantification of protein expression in Saos2 cells treated with single agent or combination BETi+CHK1i. Proteins of interest were quantified from Fig. 6 . Saos2 cells were treated with vehicle, BETi/OTX-015 (0.31μM) and/or CHK1i/SRA737 (3.75μM) for 4 hrs or 16 hrs. Vinculin served as the loading control. **(a-l)** Image Lab software (Bio-Rad) was used for protein quantification. **(a-j)** Relative protein levels were first calculated by normalizing to media-treated and vehicle-treated cells. However, there was no statistical difference observed in the data using these two different normalization methods. Therefore, proteins of interest were normalized to total protein as quantitated from the corresponding blots and expressed relative to vehicle-treated control cells. **(k-l)** Expression of phosphorylated proteins (p-CHK1 S345 and γ-H2AX) were also normalized to their total respective protein levels. This experiment was performed one time. *OTX = BETi/OTX-015, SRA = CHK1i/SRA737 , and combo= BETi/OTX-015+ SRA = CHK1i/SRA737*

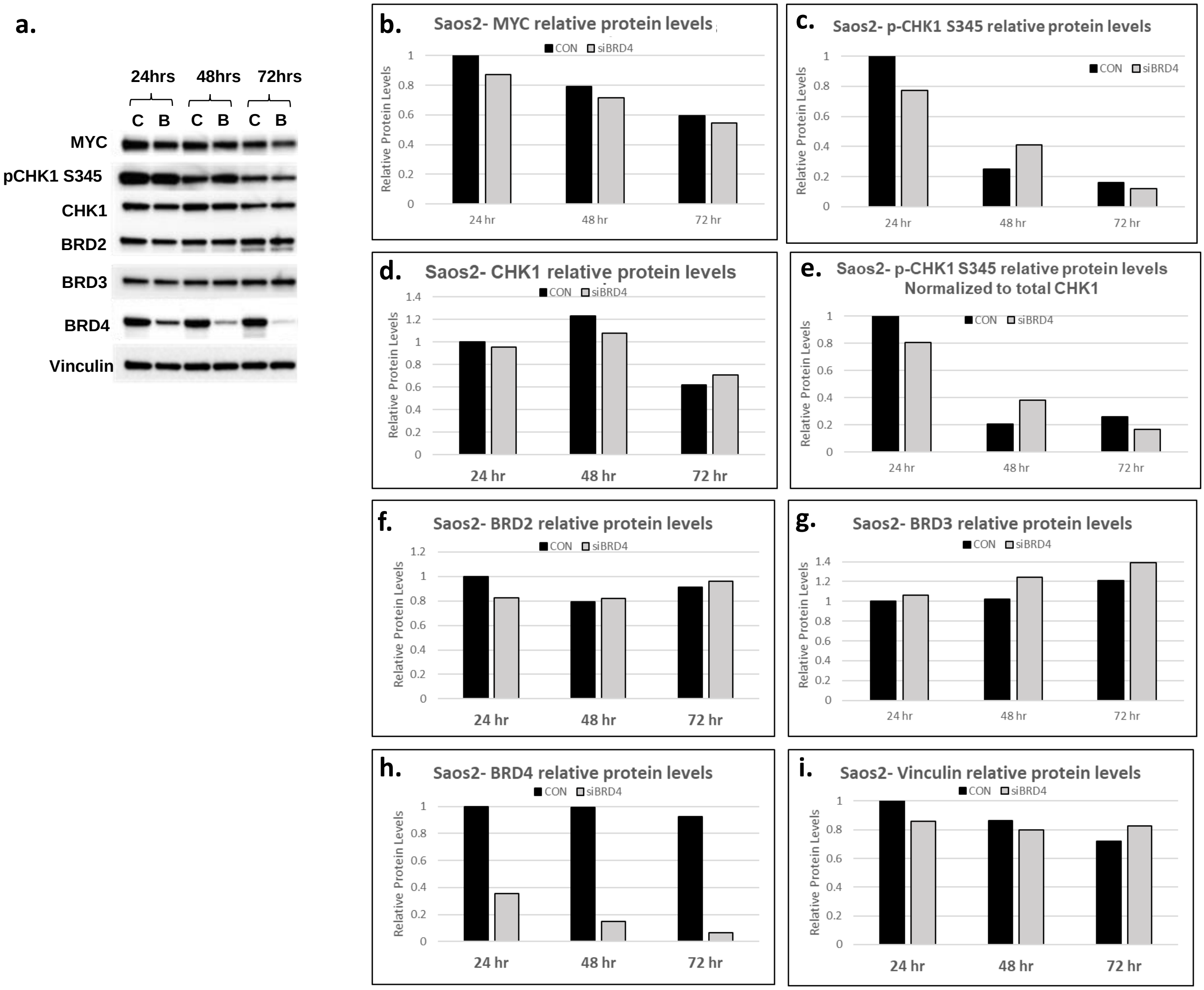

Figure S7: Knock down of BRD4 via siRNA does not decrease MYC, CHK1 or other BET proteins. **(a)** Whole cell lysates from Saos2 cells treated with 100nM control siRNA or BRD4 siRNA for 24hrs, 48hrs, and 72hrs were evaluated for expression of proteins of interest. *C= control siRNA and B = BRD4 siRNA* **(b-i)** Quantification of protein expression in control siRNA- or BRD4 siRNA-treated Saos2 cells was determined using the Image Lab software (Bio-Rad). For **Figs. S7b-d** and **Figs. S7f-i,** the proteins of interest were normalized to total protein as quantitated from the corresponding blot and expressed relative to control siRNA treated cells. For **Fig. S7e**, Expression of p-CHK1 S345 was also normalized to total CHK1 protein. *V*inculin served as the loading control. This experiment was performed one time. *CON = control siRNA and siBRD4 = BRD4 siRNA*

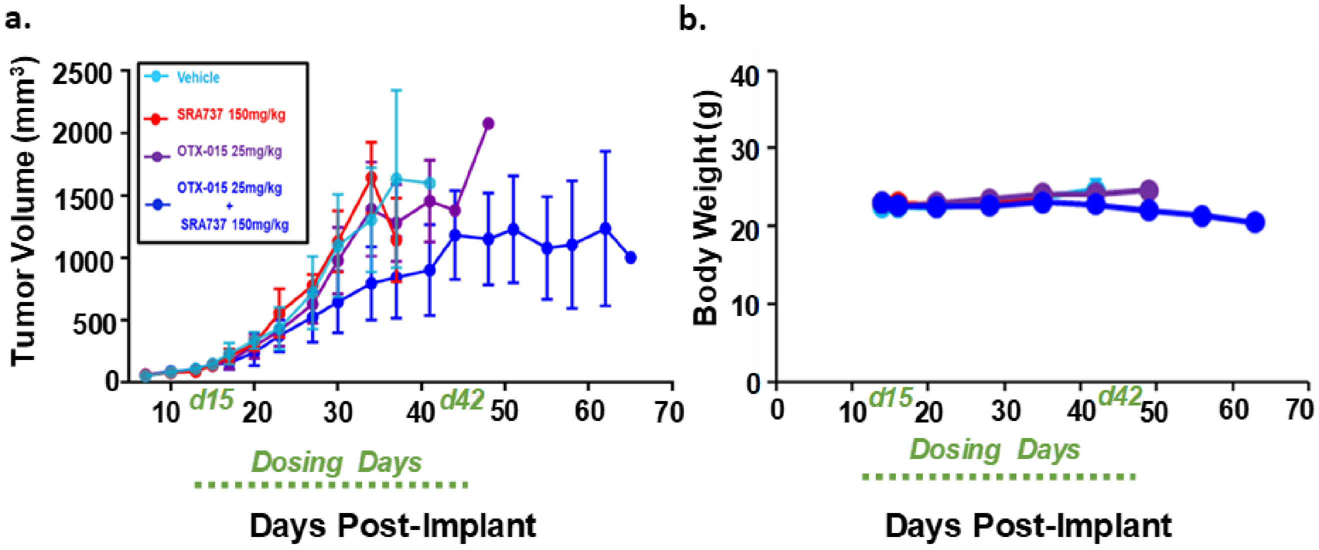

Figure S8: Dual-inhibition of BET and CHK1 effectively decreases tumor growth and is well-tolerated in the MYC-RAD21+ Saos2 CDX. (a) Tumor growth kinetics over time. (b) Body weights are maintained over time following single-agent or combination BETi + CHK1i therapy. Refer to Fig. 8 for further details.

a.

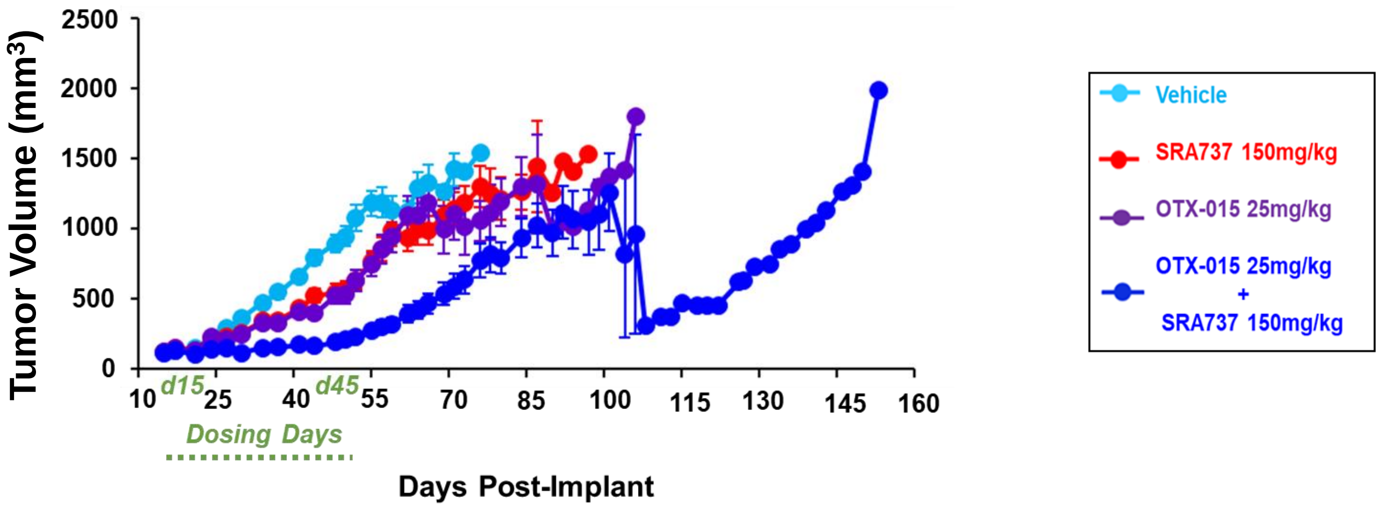

b.

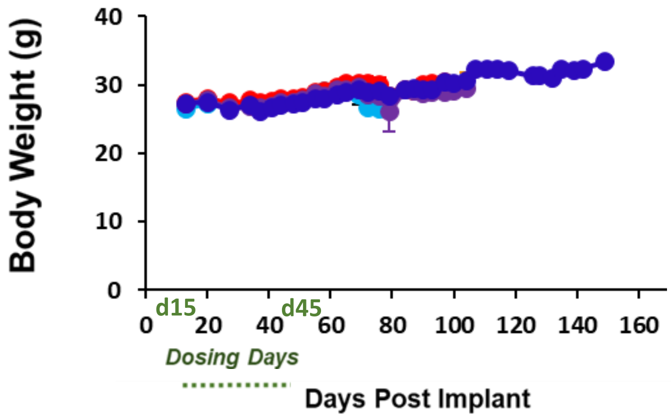

c.

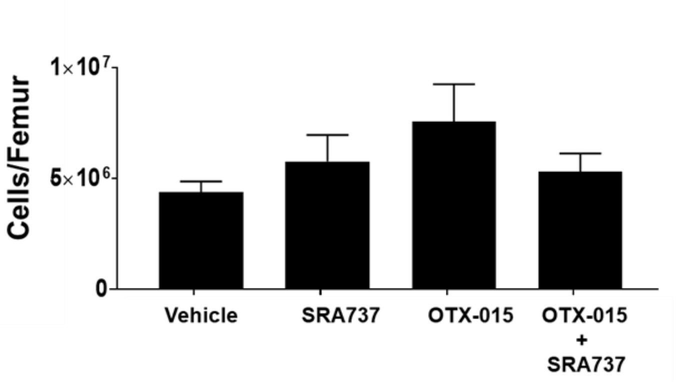

Figure S9: Inhibition of BET in combination with CHK1 inhibitor effectively decreases tumor growth kinetics and is well-tolerated in an AYA relapsed MYC-RAD21+ PDX. (a) tumor growth kinetics over time. For statistical significance during the dosing period please refer to Fig. 9a in the main manuscript (b) Body weights are maintained over time following single-agent or combination BETi + CHK1i therapy. (c) Bone marrow cellularity was determined at the end of the study (n=4 mice/group). Refer to Fig. 9 for further details.

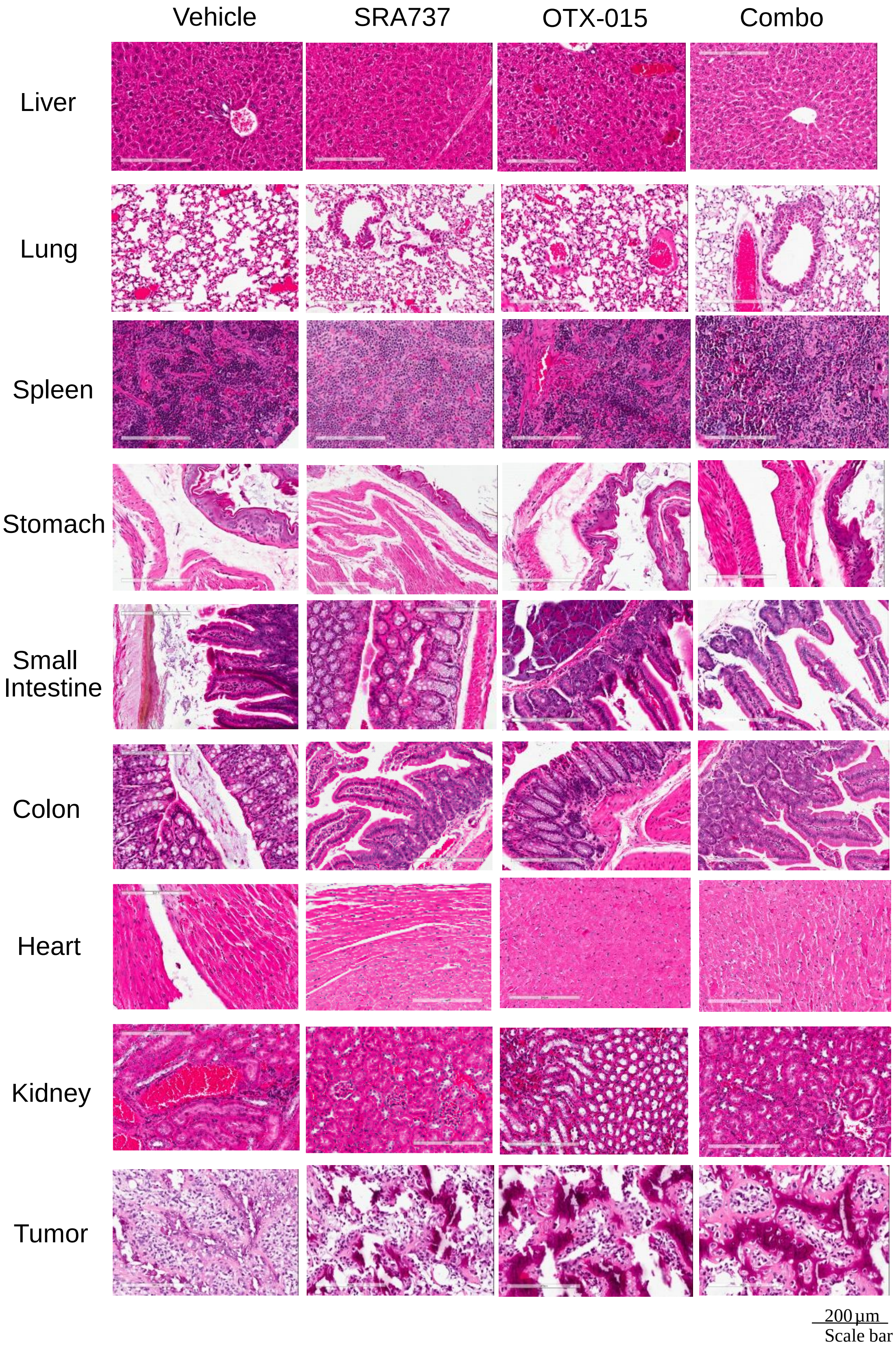

Figure S10: Single-agent or BETi + CHK1i combination therapy does not affect organ tissue integrity. At the end of the study, tissues were harvested, formalin fixed, processed and stained with H&E. Organs evaluated were the liver, lung, spleen, stomach, small intestine, colon, heart and kidney. Vehicle-treated tumors have moderate differentiated OS with some osteoid. Treatment with CHK1i / SRA737 as single agent show moderate differentiated OS with extensive mineralization in tumor. Tumors treated with BETi / OTX-015 show moderate differentiated OS with osteoid and mineralization. Combination (Combo = SRA737 + OTX-105) therapy resulted in tumors with moderate differentiated OS with osteoid and mineralization. H&E stains are representative of 3 mice per group. All images are at 20x magnification.

SUPPLEMENTAL METHODS

Western Blot Analysis (Fig. S2-S4, S6-S7): method is explained in main text – in Materials and Methods Section

Methylene Blue Staining Pictures and quantification (Fig S5): Saos2 cells (100,000 cells/well) were seeded overnight in 6-well plates. They were treated the next day with vehicle, OTX-015 0.31μM, SRA737 3.75μM, and OTX-015 0.31μM + SRA737 3.75μM. After 5 days, the cells were fixed with methanol, stained with 1 ml 0.05% methylene blue per well and pictures were taken. Methylene blue (LabChem cat# LC169202)was then 2ml of 0.5M HCl (Thermo Fisher cat# A144-500) was added to each well. Subsequently, 150ul was removed from each well to a 96-well plate to read absorbance at 660nm by plate reader (BioTek Synergy H4 machine).

Bone Marrow Cellularity For Toxicity (Fig. S9): Tibia and femurs were collected and crushed to obtain isolated cell suspensions as described by Tonsing-Carter et al [91]. The cell suspensions were filtered through 0.45 mm filters. Subsequently, red blood cells (RBC) were depleted with RBC lysis buffer (Qiagen) and total number of viable bone-marrow cells determined by trypan blue exclusion.

Analysis of tissue integrity (Fig. S10): Tissues were fixed in 10% neutral-buffered formalin at 4°C for 72 hours and then embedded in paraffin. Five μm sections were cut and flattened on a heated water bath, floated onto microscope slides and dried. For routine assessment slides were stained with hematoxylin and eosin (H&E; Harris hematoxylin, regressive method).
