# Supplementary material for: Systems Biology Approach Identifies Prognostic Signatures of Poor Overall Survival and Guides the Prioritization of Novel BET-CHK1 Combination Therapy for Osteosarcoma
Source: Cancers (Basel). 2020 Aug 26;12(9):2426. doi: 10.3390/cancers12092426 (PMC7564419; doi:10.3390/cancers12092426)
Supplement: Supplementary file 1 [file cancers-12-02426-s001.zip › Original Western Blot images.pdf]

Fig. S2

Whole blot with bands and all molecular weights

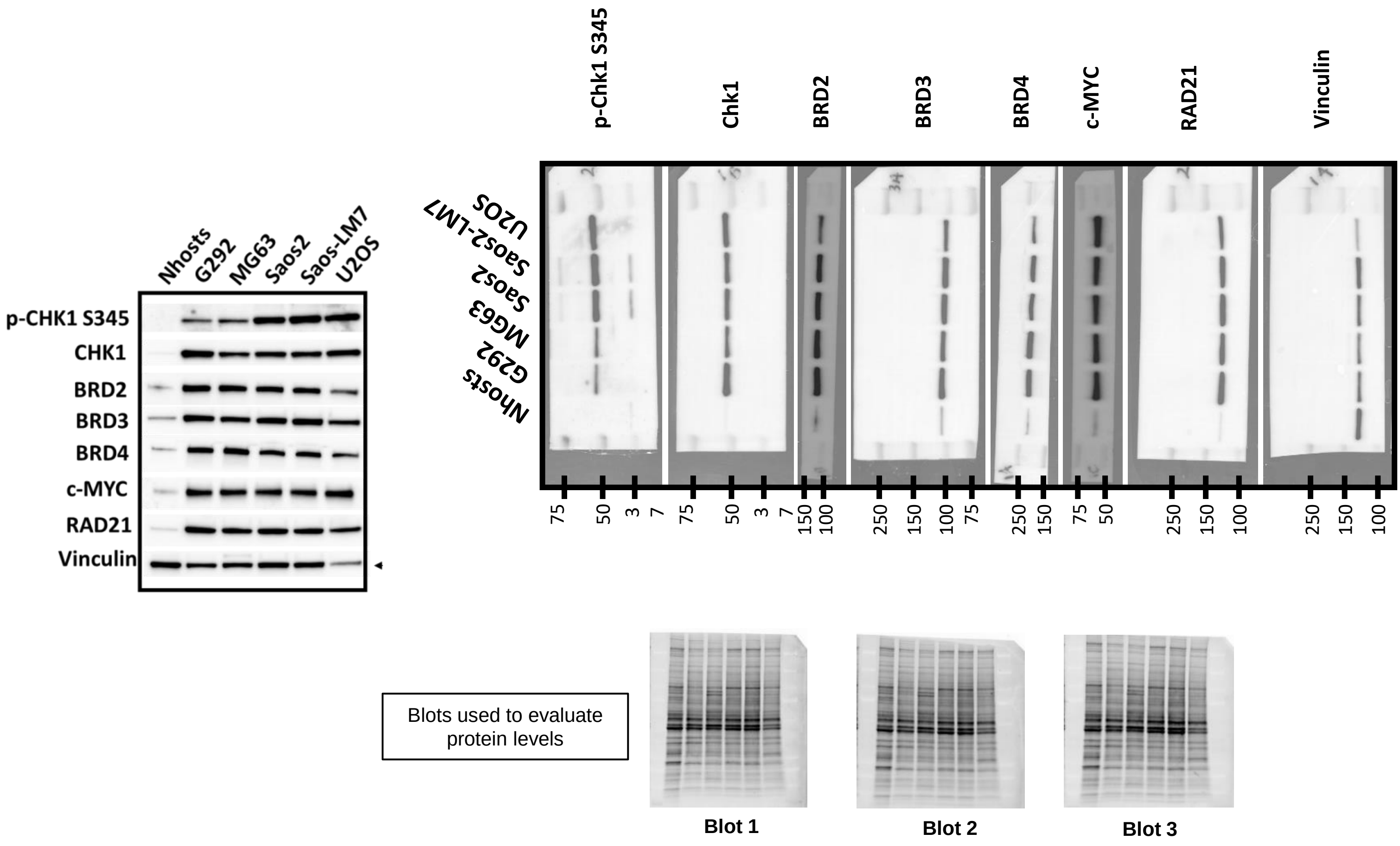

Figure S2: Validation of targets of interest in established OS cell lines. *Whole blot with bands and all molecular weights*

Fig. S3

Whole blot with bands and all molecular weights

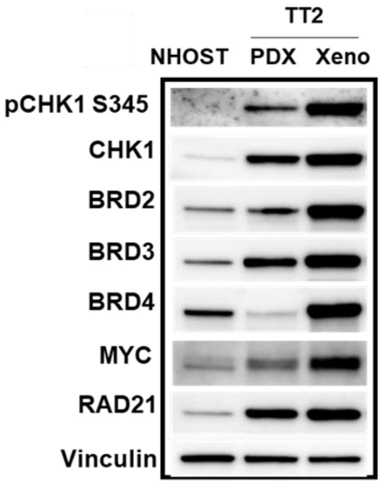

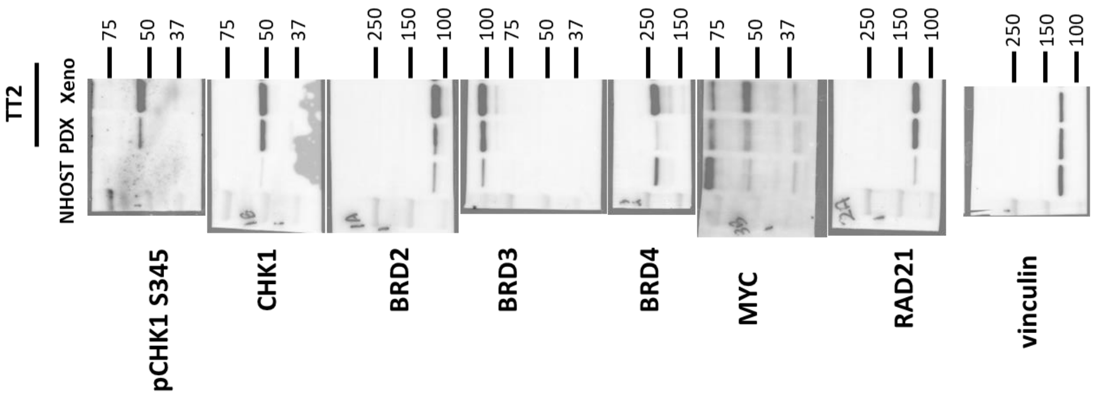

Blots used to evaluate protein levels

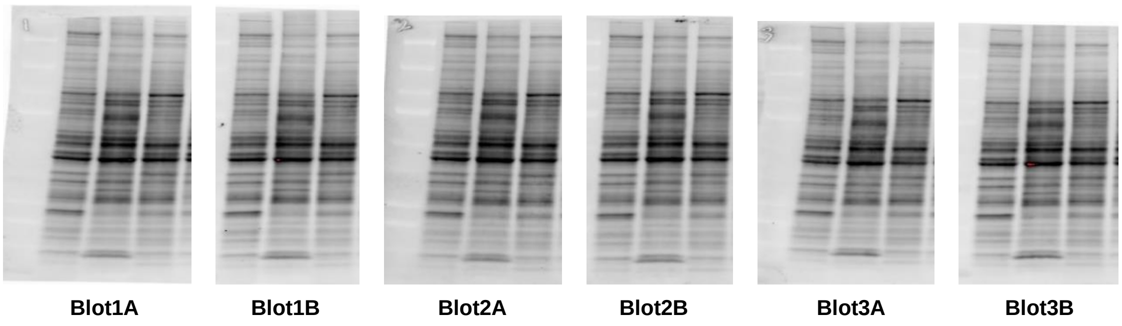

Figure S3: Validation of targets in TT2-77 PDX and PDX-derived OS. *Whole blot with bands and all molecular weights*

Fig. S4

Whole blot with bands and all molecular weights

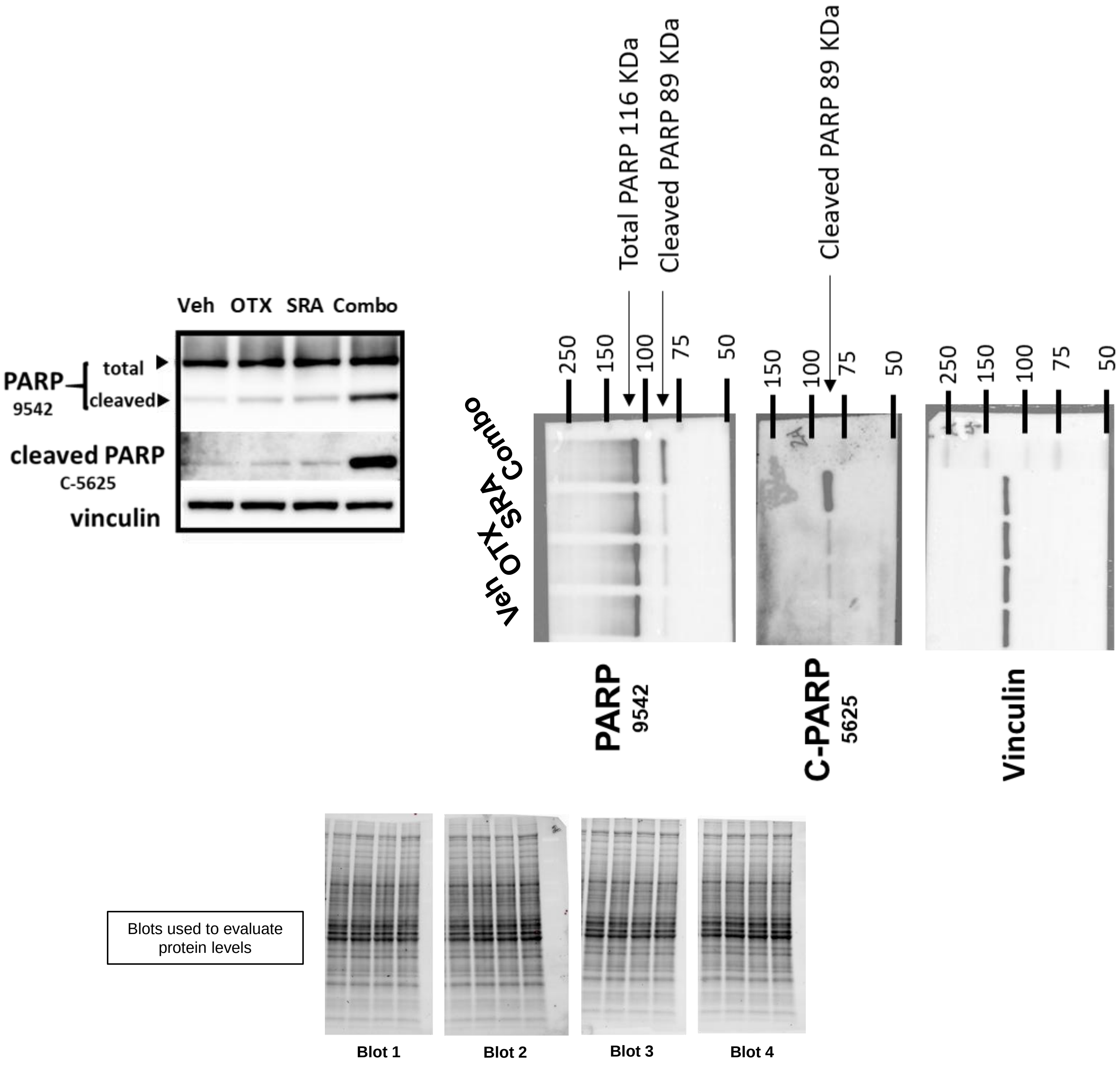

Figure S4: Quantification of cleaved –PARP protein expression in Saos2 cells treated with single agent or combination BETi + CHK1i. Proteins of interest were quantified from Fig. 5b . Veh = vehicle; OTX= BETi/OTX-015; SRA= CHK1i/SRA737; Combo = OTX (0.31µM) + CHK1i/SRA737 (3.75µM).*Whole blot with bands and all molecular weights*

Figs. S6

Whole blot with bands and all molecular weights

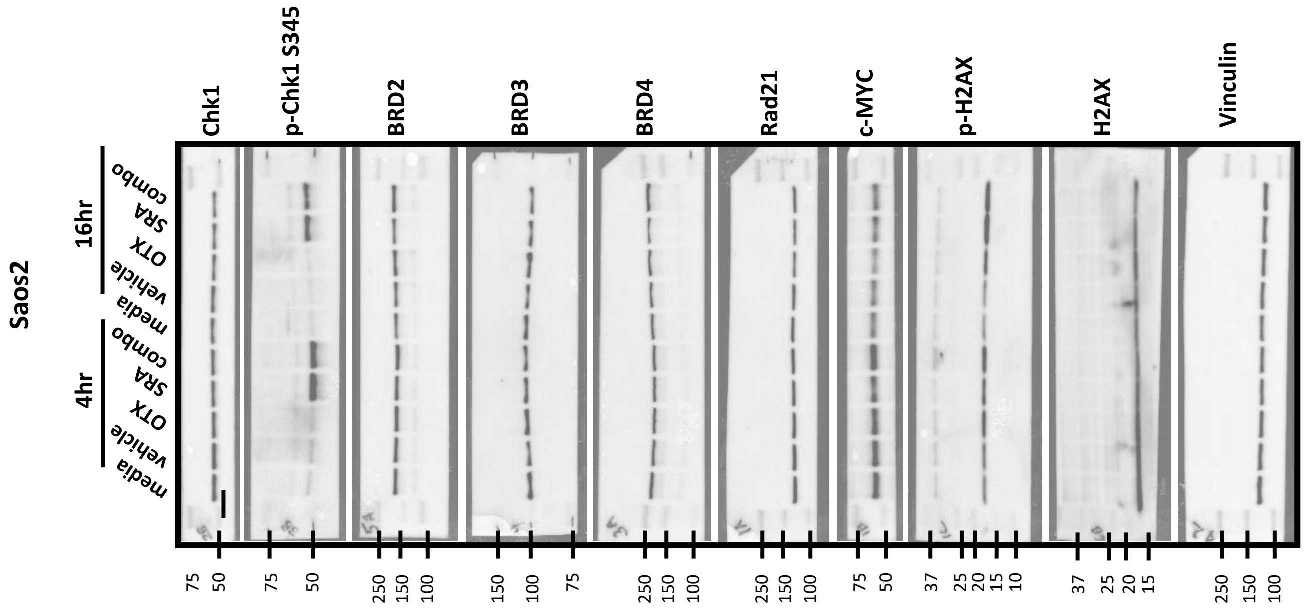

Blots used to evaluate protein levels

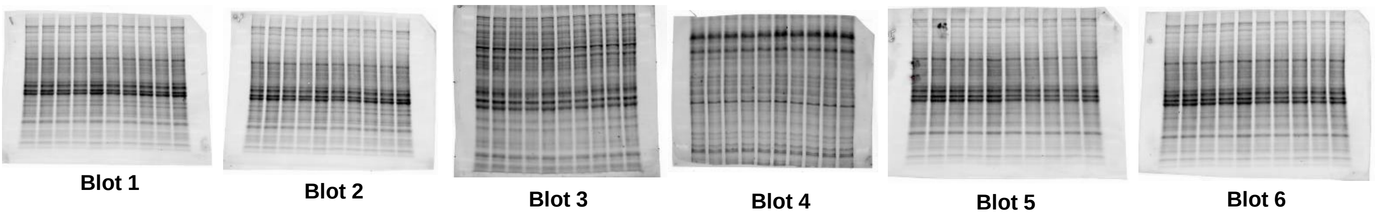

Figure S6: Quantification of protein expression in Saos2 cells treated with single agent or combination BETi+CHK1i.
Whole blot with bands and all molecular weights

Fig. S7

Whole blot with bands and all molecular weights

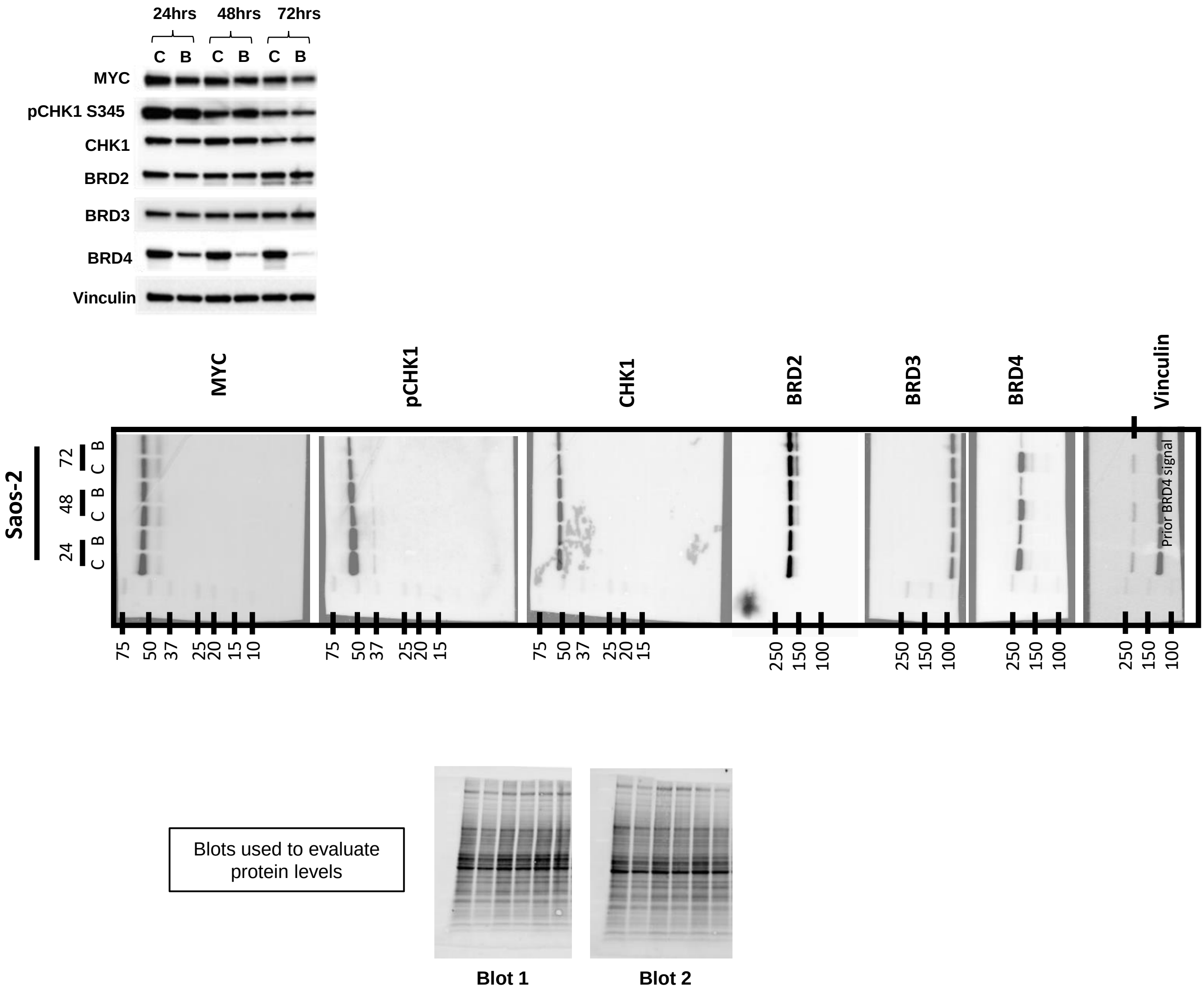

Figure S7: BRD4 siRNA treated Saos2 cells resulted in down-regulation of BRD4 but not MYC, CHK1, or other BET proteins. *C= control siRNA; B= BRD4 siRNA.* *Whole blot with bands and all molecular weights*
